# Linking Light-Dependent Life History Traits with Population Dynamics for *Prochlorococcus* and Cyanophage

**DOI:** 10.1128/mSystems.00586-19

**Published:** 2020-03-31

**Authors:** David Demory, Riyue Liu, Yue Chen, Fangxin Zhao, Ashley R. Coenen, Qinglu Zeng, Joshua S. Weitz

**Affiliations:** a School of Biological Sciences, Georgia Institute of Technology, Atlanta, Georgia, USA; b Division of Life Science, The Hong Kong University of Science and Technology, Hong Kong, China; c Department of Ocean Science, The Hong Kong University of Science and Technology, Hong Kong, China; d School of Physics, Georgia Institute of Technology, Atlanta, Georgia, USA; e HKUST Shenzhen Research Institute, Shenzhen, China; f Hong Kong Branch of Southern Marine Science and Engineering, Guangdong Laboratory (Guangzhou), HKUST, Hong Kong, China; University of British Columbia

**Keywords:** cyanobacteria, cyanophage, diurnal rhythm, light-dark cycle, modeling, virus

## Abstract

*Prochlorococcus* cyanobacteria grow in diurnal rhythms driven by diel cycles. Their ecology depends on light, nutrients, and top-down mortality processes, including lysis by viruses. Cyanophage, viruses that infect cyanobacteria, are also impacted by light. For example, the extracellular viability and intracellular infection kinetics of some cyanophage vary between light and dark conditions. Nonetheless, it remains unclear whether light-dependent viral life history traits scale up to influence population-level dynamics. Here, we examined the impact of diel forcing on both cellular- and population-scale dynamics in multiple *Prochlorococcus*-phage systems. To do so, we developed a light-driven population model, including both cellular growth and viral infection dynamics. We then tested the model against measurements of experimental infection dynamics with diel forcing to examine the extent to which population level changes in both viral and host abundances could be explained by light-dependent life history traits. Model-data integration reveals that light-dependent adsorption can improve fits to population dynamics for some virus-host pairs. However, light-dependent variation alone does not fully explain realized host and virus population dynamics. Instead, we show evidence consistent with lysis saturation at relatively high virus-to-cell ratios. Altogether, our study represents a quantitative approach to integrate mechanistic models to reconcile *Prochlorococcus*-virus dynamics spanning cellular-to-population scales.

**IMPORTANCE** The cyanobacterium *Prochlorococcus* is an essential member of global ocean ecosystems. Light rhythms drive *Prochlorococcus* photosynthesis, ecology, and interactions with potentially lethal viruses. At present, the impact of light on *Prochlorococcus*-virus interactions is not well understood. Here, we analyzed *Prochlorococcus* and virus population dynamics with a light-driven population model and compared our results with experimental data. Our approach revealed that light profoundly drives both cellular- and population-level dynamics for some host-virus systems. However, we also found that additional mechanisms, including lysis saturation, are required to explain observed host-virus dynamics at the population scale. This study provides the basis for future work to understand the intertwined fates of *Prochlorococcus* and associated viruses in the surface ocean.

## INTRODUCTION

The unicellular cyanobacterium *Prochlorococcus* dominates the phytoplankton community and is a major contributor to primary production in tropical and subtropical oligotrophic oceans (1). The ecology of *Prochlorococcus* is a function of physicochemical properties of the marine environment (2–4), bottom-up forces (i.e., nutrient driven), and top-down (i.e., mortality-driven) effects (5–11). Among top-down factors, cyanophage (i.e., viruses that infect cyanobacteria) are highly abundant and can drive up to 30% of cyanobacterial mortality in marine environments (12–17). Light, temperature, and nutrients influence *Prochlorococcus* growth (2, 3, 18), as well as its interactions with cyanophage (19).

*Prochlorococcus* cyanobacteria are distributed across temperature and light gradients in the ocean environment (3, 20–22). They are specialized into high-light (HL)- and low-light (LL)-adapted ecotypes (23–25). LL ecotypes have a high fluorescence and exhibit photoinhibited growth at medium light intensity. They grow faster at low irradiance with a high concentration of divinyl chlorophyll *a* and *b* and have several *pcb* genes encoding constitutive photosystems I and II (23–25). In contrast, HL ecotypes grow faster at medium light intensities, have a low concentration of divinyl chlorophyll *a* and *b*, and have only constitutive photosystem II light-harvesting complexes (23–25). *Prochlorococcus* cells do not have a circadian rhythm; rather, they have a diurnal rhythm that can be synchronized under light-dark cycles (21, 26, 27). This diurnal rhythm is divided predominantly into photosynthesis during the light phase and cell division associated with energy consumption during the dark phase (22, 28).

Cyanophage are also impacted by light. UV radiation can deactivate and degrade virus particles (19), as well as degrade and modify viral proteins and genomes (29, 30). Light can also affect viral interactions with host cells. Previous studies suggested a dependence of viral production on the host cell cycle in different phytoplankton lineages (31–34). In contrast, the intracellular production of some viruses may be decoupled from host cell cycle and light levels (35, 36). A recent paper on the diel infection pattern of cyanophage infecting *Prochlorococcus* (37) suggests that adsorption, as well as the transcription rhythm of cyanophage, may be related to the light-dark cycle and not to the host cell cycle. Analyzing light impacts on cyanophage-cyanobacterium dynamics requires some elaboration of the cyanophage life cycle.

**FIG 1:**
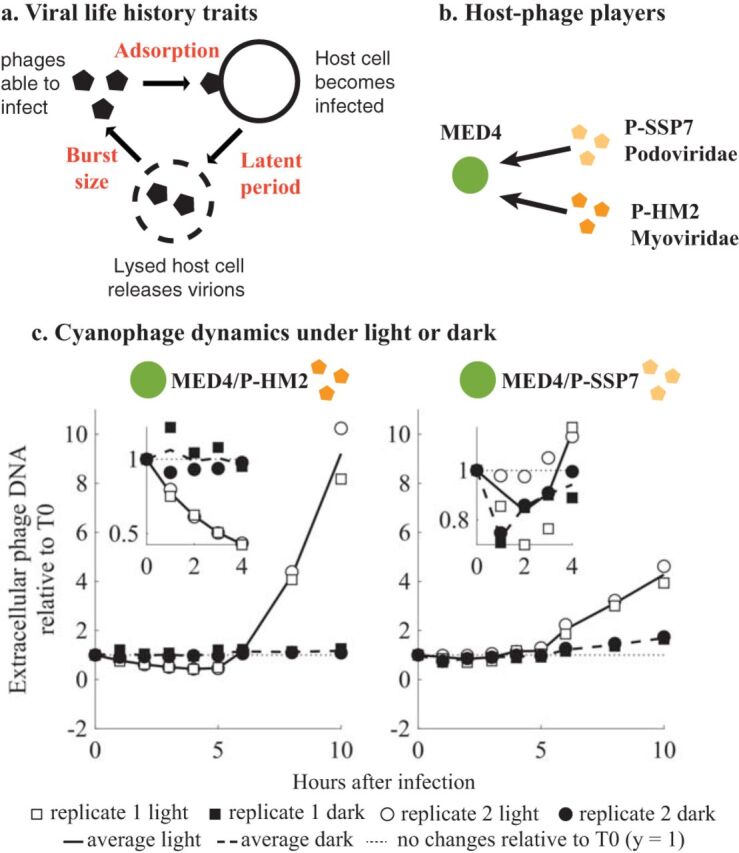
Cyanophage infection in the light or the dark. (a) Viral life history trait definitions: viral adsorption (encounter and adsorption on a noninfected host, in ml h^−1^), latent period (time between adsorption and lysis of the host cell, in hours), and burst size (new phages produced per one lysed host cell). (b) Host-cyanophage pairs used in the study. (c) Infection under light or dark (data from reference 37; see Materials and Methods). Cyanophage P-HM2 and P-SSP7 were used to infect their host cells under continuous light or in the dark. For all the host-phage pairs, the phage/host ratio is 0.1. Extracellular phage concentrations were measured as phage DNA by quantitative PCR and normalized to the value at time zero.

The lytic cyanophage cycle can be summarized into three phases: the adsorption phase, where virions attach to their host and inject their genetic material into the host cell, the cyanophage replication phase, and the lytic phase, where new virus particles are released by lysing their host (Fig. 1a). Light can affect each of these phases and associated viral life history traits (LHTs). A study on *Synechococcus* infection showed a significant decrease in adsorption under the dark condition for some phages (36, 38, 39), whereas other cyanophage adsorb during light or dark conditions (36). Similarly, light conditions also modify the cyanophage replication phase. An increase in viral production in light and a reduction in viral production in dark have been reported for *Synechococcus* (39–41) and *Prochlorococcus* (42–44).

A salient example of light-driven changes to viral LHTs is found in Liu et al. (37), who investigated infection dynamics for cyanophage infecting *Prochlorococcus* under light-dark cycles (Fig. 1b and c). The results suggest that cyanophage strains respond differently to light or dark conditions (Fig. 1c). Infection under light was always efficient for all strains. However, P-SSP7 could infect and produce viruses in the dark, while P-HM2 could not adsorb to hosts or produce viruses in the dark. These observations under fixed light or dark conditions form the central motivation for our study. Here we ask: do differences in the response of viruses to light conditions at the cellular level explain population-level dynamics of both *Prochlorococcus* and cyanophage given diurnal rhythms of light-dark cycles?

To answer this question, we couple mathematical models, high-resolution (i.e., subdaily) measurements, and model-data integration to explore the interactions between *Prochlorococcus* strain MED4 (an HL ecotype) and cyanophage P-HM2 and P-SSP7 (Fig. 1b). The bulk of data used in this study have been presented elsewhere (see data attribution statements) (37, 45). The models extend the framework of nonlinear population dynamics of lytic viruses and their hosts (46) to an explicitly light-driven context (see the related work of reference 47 on coccolithoviruses and their Emiliana huxleyi hosts given daily measurements). As we show, although diel-driven viral life history traits help explain population dynamics, they are not necessarily sufficient. Instead, our study identifies additional mechanisms involving saturating lysis that help reconcile population-level dynamics of cyanophage and cyanobacteria.

## RESULTS

### Light-driven *Prochlorococcus* growth.

We first estimated the growth of *Prochlorococcus* strain MED4 in culture under light-dark cycles and nutrient-nonlimited conditions during the exponential-growth phase. We used an ordinary differential equation (ODE) model to describe the dynamics of the *Prochlorococcus* population (*P*) (cells/ml), as follows:

(1) $$\dot{P}=(\mu -\omega )P$$

where ω is the host mortality rate (h^−1^) and *μ* is the host light-derived growth rate (h^−1^) as a function of perceived light during the experiments (48), as follows:

(2) $$\mu (L_{t})=\mu_{\text{opt}}\frac{L_{t}^{4}}{L_{t}^{4}+K_{L}^{4}}$$

Here, the host growth rate increases nonlinearly with the amount of light, following a Hill function. *K_L_* is the minimum amount of light necessary for a cell to divide (in μmol s^−1^ m^−2^), and *L_t_* is the cumulative light perceived by a cell at time *t* (in μmol s^−1^ m^−2^) depending on the light-dark cycle state, as follows:

(3) $$L_{t}=\{\begin{array}{ll} L\tau , & \text{during light phase}\\ L\left[n_{\text{light}}-(\tau -n_{\text{dark}})\right]\left(\frac{n_{\text{light}}}{n_{\text{dark}}}\right), & \text{during dark phase} \end{array}$$

where τ is equal to the remainder of the division of the time *t* by 24 h [formally, τ = rem(*t*,24)], *n*_light_ and *n*_dark_ are the number of hours of light and dark during the cycle, respectively, and *L* is the irradiance during the light phase of the experiments (μmol s^−1^ m^−2^). μ_opt_ is the optimal host growth rate (*h*^−1^) defined by the growth-irradiance function described in reference 49 with the following equation:

(4) $$\mu_{\text{opt}}(L)=\mu_{\text{max}}\frac{L}{L+\frac{\mu_{\text{max}}}{\alpha }{\left(\frac{L}{L_{\text{opt}}}-1\right)}^{2}}$$

In this functional form, μ_max_ is the maximal host growth rate (*h*^−1^) at optimal light *L*_opt_ (μmol s^−1^ m^−2^), and α is the initial slope of the light response curve (*h*^−1^). During the 24 h of a light-dark cycle, μ(*L_t_*) increases from 0 during the first 14 h (light period) to reach a maximum after *n*_light_ h of light and then decreases during the *n*_dark_ h of the dark period.

**FIG 2:**
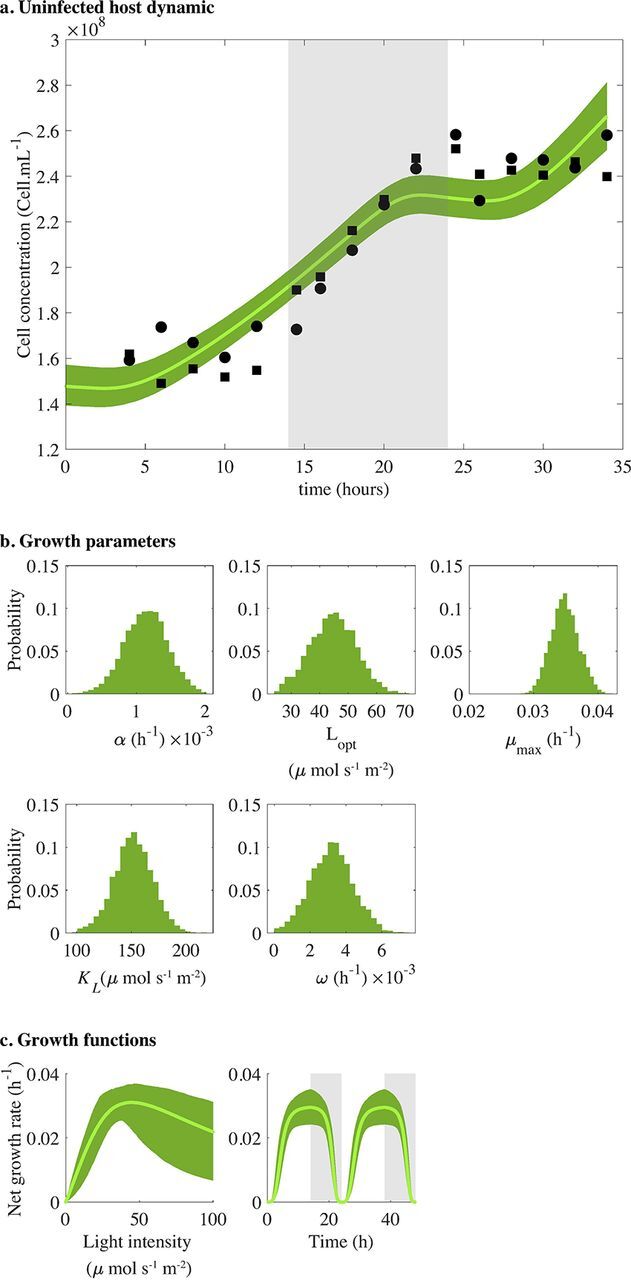
Modeling *Prochlorococcus* MED4 strain as function of light without viruses during the exponential phase. (a) Fit of the host dynamic (equation 1). Solid lines represent the median of 5,000 model simulations, and shaded areas are the 95% quantiles. Black dots are data (from reference 45) for two replicates, and gray shaded area represents the dark condition. (b) Model growth parameter distributions of the host model (equations 1 and 4). Parameter distribution estimated using an MCMC algorithm: photosynthesis-irradiance (PI)-curve slope of the linear phase *α*, optimal growth light *L*_opt_, maximal growth rate *μ*_max_, minimum amount of light necessary to divide *K_L_*, and natural mortality *ω*. (c) Model growth functions that drive the host dynamic: growth is expressed as the net growth rate (μ_opt_ − ω) as a function of irradiance (equation 4; left) and as a function of time (equation 2; right).

The model in equation 1 was fit to population abundance measurements of *Prochlorococcus* strain MED4 under a light-dark cycle (45) using a Markov chain Monte Carlo (MCMC) approach (see Materials and Methods). The best-fit light-driven host growth model recapitulates the experimental data (Fig. 2a) with a good convergence of the MCMC parameter chains (Fig. S1a and Table S1 in the supplemental material). MED4 has a low growth-irradiance curve slope (α = 0.0011 h^−1^), a high optimal growth irradiance (*L*_opt_ = 44.78 μmol s^−1^ m^−2^), and a maximum growth rate of 0.0035 h^−1^ (Fig. 2b and Fig. S1a). These parameters are consistent with prior estimates of HL growth-irradiance characteristics for strain MED4 (Fig. 2c) (50).

10.1128/mSystems.00586-19.1FIG S1Chain convergences for the host growth parameters for *Prochlorococcus* strain MED4 (a), for the infection parameters of P-HM2 infecting strain MED4 for the best hypothesis $\tilde{H1}$ (b), and for the infection parameters of P-SSP7 infecting strain MED4 for best hypothesis $\tilde{H0}$ (c). Download REVISED FIG S1, PDF file, 1.2 MB [ORIGINAL FIG S1, PDF file, 1.2 MB].Copyright © 2020 Demory et al.2020Demory et al.This content is distributed under the terms of the Creative Commons Attribution 4.0 International license.

10.1128/mSystems.00586-19.7 TABLE S1 Host growth parameter means and standard deviations. Download Table S1, PDF file, 0.05 MB. Copyright © 2020 Demory et al. 2020 Demory et al. This content is distributed under the terms of the Creative Commons Attribution 4.0 International license.

### Modeling *Prochlorococcus*-phage dynamics under light-dark cycles.

**FIG 3:**
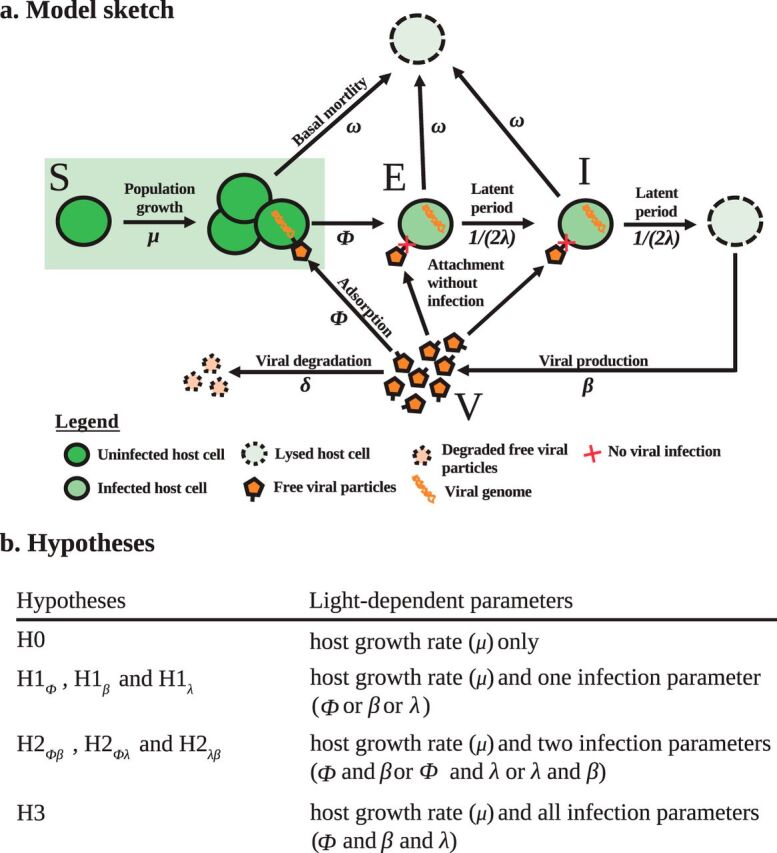
Description of the model. (a) Schematic representation of the model. The host population is divided into 3 classes: susceptible (S), exposed (E), and infected (I) types. The virus particle density is denoted by *V*. Black arrows are biological processes. (b) Definitions of the hypotheses. Each hypothesis describes a possible relation between light and the infection parameters. When parameter ϕ, β, or λ is light dependent, it is a piecewise function, i.e., it takes one value in light and one value in dark.

To investigate *Prochlorococcus*-cyanophage dynamics under light-dark cycles, we developed a nonlinear ODE population model describing the infection of *Prochlorococcus* by cyanophage (Fig. 3a), extending existing frameworks for modeling obligately lytic phage-host interactions (46). We used a two-stage infection model to account for the finite latent period of the infection. The host population is categorized as susceptible (*S*), exposed (*E*), and infected (*I*), such that the total host population is *N* = *S* + *E* + *I*. The density of free cyanophage is denoted by *V*. The model is described by the following system:

(5) $$\begin{array}{lll} \overset{˙}{S} & = & \overset{\text{Host growth}}{\overset{︷}{\mu S\left(1-\frac{N}{K}\right)}}-\overset{\text{Basal loss}}{\overset{︷}{\omega S}}-\overset{\text{Viral adsorption}}{\overset{︷}{\phi \mathrm{SV}}},\\ \overset{˙}{E} & = & \overset{\text{Exposed}}{\overset{︷}{\phi \mathrm{SV}}}-\overset{\text{Basal loss}}{\overset{︷}{\omega E}}-\overset{\text{Active infection}}{\overset{︷}{\frac{1}{2\lambda }E}},\\ \overset{˙}{I} & = & \overset{\text{Active infection}}{\overset{︷}{\frac{1}{2\lambda }E}}-\overset{\text{Basal loss}}{\overset{︷}{\omega I}}-\overset{\text{Lysis}}{\overset{︷}{\frac{1}{2\lambda }I}},\\ \overset{˙}{V} & = & \overset{\text{Lysis}}{\overset{︷}{\frac{\beta }{2\lambda }I}}-\overset{\text{Viral attachment}}{\overset{︷}{\phi \mathrm{NV}}}-\overset{\text{Virion decay}}{\overset{︷}{\delta V}} \end{array}$$

In this model, μ is the host growth rate (*h*^−1^), *K* is the host carrying capacity (cells ml^−1^), ω is the host basal mortality (*h*^−1^) not due to viral lysis, ϕ is the adsorption rate (ml *h*^−1^), λ is the average latent period (*h*), β is the burst size (unitless), and δ is the viral decay rate (*h*^−1^) (see Table S2 for more information on parameters). We assume that viruses can attach to all host cells (*S*, *E*, and *I*) but only lead to state transitions when infecting *S* types, i.e., from susceptible to exposed. We have already established that light modulates host growth (Fig. 2). However, it is not evident whether diel variation in host growth alone can explain changes in virus and host dynamics at population scales. Hence, we defined a series of nested model hypotheses that include alternative mechanisms for light-driven changes in viral life history traits (Fig. 3b). The mechanisms are different in the number of viral life history traits that differ between light and dark. The number ranges from 0 (in the null hypothesis *H*0) to 3 (*H*3), where the adsorption rate, latent period, and burst size each differ between light and dark. In practice, each model parameter that is light driven takes on two values in the model, e.g., the burst size would have β_dark_ and β_light_. Although viruses are known to be degraded under UV light (19), our experiments were conducted under white light without UV radiation and viral decay rates were similar under light or dark conditions (Fig. S2). Hence, we fixed the value of decay rates.

10.1128/mSystems.00586-19.2 FIG S2 Experimental estimation of viral decay under light and dark conditions for P-HM2 and P-SSP7. Download FIG S2, PDF file, 0.2 MB. Copyright © 2020 Demory et al. 2020 Demory et al. This content is distributed under the terms of the Creative Commons Attribution 4.0 International license.

10.1128/mSystems.00586-19.8 TABLE S2 Description of model parameters. Download Revised Table S2, PDF file, 0.03 MB [Original Table S2, PDF file, 0.03 MB]. Copyright © 2020 Demory et al. 2020 Demory et al. This content is distributed under the terms of the Creative Commons Attribution 4.0 International license.

**FIG 4:**
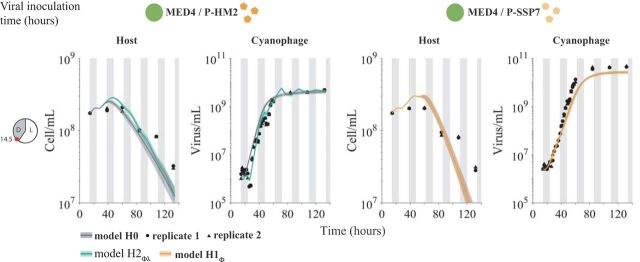
Light-driven models fit to host and virus population abundance data. Model fits under *H*0 and hypotheses *H*2_ϕλ_ and *H*1_ϕ_ for an inoculation time of 14.5 h after the beginning of the experiment. Phage P-HM2 infecting strain MED4 (left) and P-SSP7 infecting MED4 (right). Solid lines represent the median values of 5,000 model simulations, with shaded areas the 95% quantiles. Data are represented by the black dots for two replicates. Vertical shaded gray lines represent dark conditions.

We fit each of the nested, light-driven virus-host population models, using MCMC, to experimental measurements of *Prochlorococcus* strain MED4 infected by either cyanophage P-HM2 or P-SSP7 over a 4-day period (Fig. 4). Parameter ranges in the MCMC procedure were constrained by prior estimates (Tables S3 and S4) (51). We found the best-fit model while accounting for the inclusion of additional model complexity to be *H*2_ϕλ_ for P-HM2 and *H*0 for P-SSP7 (Fig. S3). This suggests that P-HM2 (but not P-SSP7) has light-dependent life history traits that help provide explanatory power to the virus-host population dynamics. The best-fitting model suggests that both adsorption and production for P-HM2 are significantly reduced in the dark compared to its adsorption and production under light conditions. In both cases, viral abundances rapidly increase and then plateau. However, in both cases, the best-fit model significantly overestimates the degree of virally induced mortality in the host population, e.g., models predict that the final time point estimates of cell density are 2.5 and 6.1 times lower than the values measured for the P-HM2 and P-SSP7 cases, respectively. This result suggests that other features underlying interactions between cyanophage and *Prochlorococcus* have to be accounted for when scaling up to the population-level dynamics.

10.1128/mSystems.00586-19.3 FIG S3 Model comparison criterion. Akaike information criteria (AIC; left panels) and Bayesian information criteria (BIC; right panels) calculated for the two host-virus pairs used in the study. Box plots are calculated over 5,000 parameter sets for each hypothesis and model. Minimum values of AIC and BIC considering the best hypotheses. Red circles indicate the best hypotheses for each host-virus pair. Download FIG S3, PDF file, 0.6 MB. Copyright © 2020 Demory et al. 2020 Demory et al. This content is distributed under the terms of the Creative Commons Attribution 4.0 International license.

10.1128/mSystems.00586-19.9 TABLE S3 P-HM2 and P-SSP7 viral life history traits measured experimentally from this study with data in Fig. 1 (see Materials and Methods) and from prior work (51). Download Table S3, PDF file, 0.04 MB. Copyright © 2020 Demory et al. 2020 Demory et al. This content is distributed under the terms of the Creative Commons Attribution 4.0 International license.

10.1128/mSystems.00586-19.10 TABLE S4 Statistics: median and quantiles (25% to 75%) of the parameter distributions for best initial and lysis inhibition model hypotheses for virus P-HM2 and P-SSP7 infecting *Prochlorococcus* strain MED4, calculated with 5,000 parameter sets. Download Revised Table S4, PDF file, 0.1 MB [Original Table S4, PDF file, 0.1 MB]. Copyright © 2020 Demory et al. 2020 Demory et al. This content is distributed under the terms of the Creative Commons Attribution 4.0 International license.

### Beyond light: incorporating lysis inhibition to explain virus and host population dynamics.

The observation that host populations remain persistently above model expectations when viral abundances are high suggests a potential slowdown mechanism in virally induced lysis. To account for this, we modified the initial model to account for an additional state transition, i.e., from *I* to *E*, as follows:

(6) $$\begin{array}{lll} \overset{˙}{S} & = & \overset{\text{Host growth}}{\overset{︷}{\mu S\left(1-\frac{N}{K}\right)}}-\overset{\text{Basal loss}}{\overset{︷}{\omega S}}-\overset{\text{Viral adsorption}}{\overset{︷}{\phi \mathrm{SV}}},\\ \overset{˙}{E} & = & \overset{\text{Exposed}}{\overset{︷}{\phi \mathrm{SV}}}-\overset{\text{Basal loss}}{\overset{︷}{\omega E}}-\overset{\text{Active infection}}{\overset{︷}{\frac{1}{2\lambda }E}}+\overset{\text{Lysis inhibition}}{\overset{︷}{\phi \mathrm{IV}}},\\ \overset{˙}{I} & = & \overset{\text{Active infection}}{\overset{︷}{\frac{1}{2\lambda }E}}-\overset{\text{Basal loss}}{\overset{︷}{\omega I}}-\overset{\text{Lysis}}{\overset{︷}{\frac{1}{2\lambda }I}}-\overset{\text{Lysis inhibition}}{\overset{︷}{\phi \mathrm{IV}}},\\ \overset{˙}{V} & = & \overset{\text{Lysis}}{\overset{︷}{\frac{\beta }{2\lambda }I}}-\overset{\text{Viral attachment}}{\overset{︷}{\phi \mathrm{NV}}}-\overset{\text{Virion decay}}{\overset{︷}{\delta V}} \end{array}$$

In this model, free virions switch the state of infection from *I* to *E*, thereby slowing down the expected time to lysis. This slowdown occurs in a fraction ϕ*V*/[1/(2λ) + ω + ϕ*V*] of cells in the *I* state; hence, it increases with increasing virus density. For example, given the best-fit parameters for P-SSP7, this fraction changes from 1.28 10^−4^ when *V *= 10^6^ virions/ml to 1.26 10^−2^ when *V *= 10^8^ virions/ml, nearly a 100-fold difference. We denote equation 6 the lysis inhibition model.

**FIG 5:**
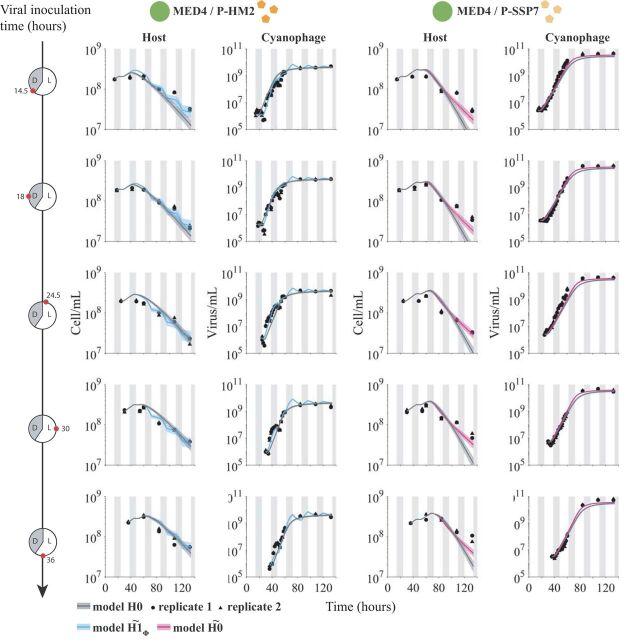
Viral dynamics under light-dark cycle for different viral inoculation times. Model fits under *H*0 and best hypotheses $\tilde{H0}$ or $\tilde{H1_{\phi }}$ for different viral inoculation times. Phage P-HM2 infecting strain MED4 (left) and P-SSP7 infecting MED4 (right). Solid lines represent the median values of 5,000 model simulations, with shaded areas the 95% quantiles. Data are represented by the black dots for two replicates. Vertical shaded gray lines represent dark conditions.

We then fit the lysis inhibition model to an expanded set of experimental measurements of MED4 infected by either cyanophage P-HM2 or P-SSP7. The measurements comprise additional time courses for each cyanophage. The time courses have different virus inoculation times of 14.5, 18, 24.5, 30, and 36 h (Fig. 5). The light-dependent hypotheses used in fitting are denoted $\tilde{H}$ to distinguish them from the original hypotheses. Via an MCMC fitting procedure, we find that the models $\overset{˜}{H1_{\phi }}$ and $\tilde{H0}$ best fit the host and virus dynamics in the P-HM2 and P-SSP7 cases, respectively (Fig. 5). Notably, the best-fit model simulations are now able to reproduce both the viral saturation and the slowdown of the host population (Fig. S4a and b). Moreover, examination of P-HM2 dynamics at the daily scale reveals differences between the light-driven viral life history traits model $\overset{˜}{H1}$ and the light-driven growth-only model *H*0 (Fig. S4c). A full list of Akaike information criterion (AIC) and Bayesian information criterion (BIC) information is found in Fig. S3. Specifically, both P-HM2 and P-SSP7 can adsorb, replicate, and lyse cells in the light. However, models suggest that P-HM2 has markedly different light-versus-dark infection life history traits, whereas there is not enough evidence to reject the null hypothesis in the case of P-SSP7.

10.1128/mSystems.00586-19.4FIG S4Zoomed-in view of the dynamics of the host MED4 infected by P-HM2 (a) and P-SSP7 (b). Solid gray line is the *H*0 model, solid blue line is the $\tilde{H1_{\phi }}$ model, and solid pink line is the $\tilde{H0}$ model. (c) Viral dynamics of the 30-h inoculation treatments for the MED4/P-HM2 pair. Black dots are the experimental data. Blue line is the light-driven viral life history traits model ($\tilde{H1_{\phi }}$), and gray line is the light-driven growth-only model (*H*0). Download FIG S4, PDF file, 0.8 MB.Copyright © 2020 Demory et al.2020Demory et al.This content is distributed under the terms of the Creative Commons Attribution 4.0 International license.

**FIG 6:**
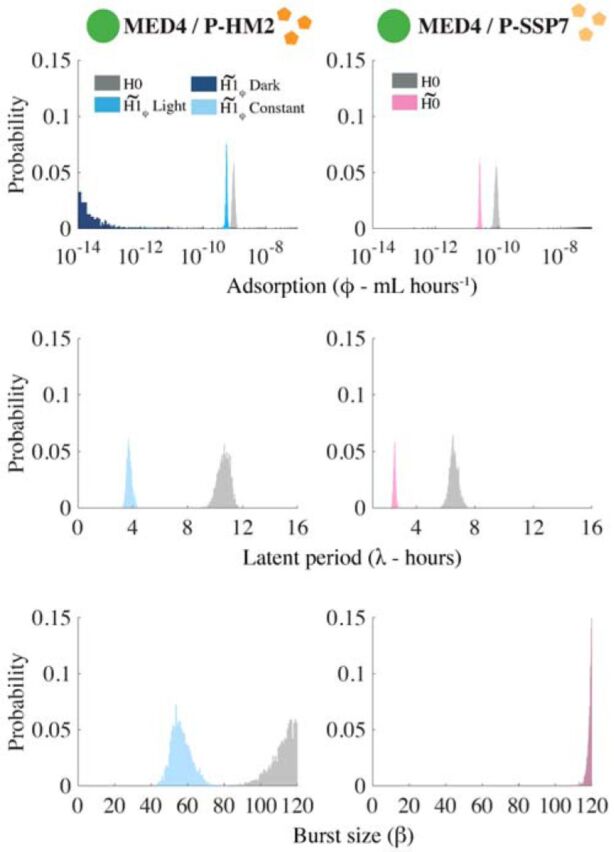
Model infection parameter distributions: P-HM2 (left) and P-SSP7 (right) infecting strain MED4. Distributions are calculated with 5,000 parameter sets. Distributions are colored according to the model hypothesis: *H*0 (gray), $\tilde{H1_{\phi }}$ (shades of blue), and $\tilde{H0}$ (pink). For the P-HM2/MED4 hypothesis $\tilde{H1_{\phi }}$, ϕ is light dependent, while λ and β are not (Constant; light blue). The light-dependent ϕ can take two values: one during light (Light; medium blue) and one during dark (Dark; dark blue).

We evaluated the quality of fits by assessing the predicted estimates of life history traits for the P-HM2 and P-SSP7 cases. Disparities in parameters under light or dark conditions obtained with our MCMC approach are consistent with earlier measurements of viral infections of MED4 given fixed light or dark conditions over a 10-h period (37). Specifically, model fits reveal that P-HM2 has a significantly lower adsorption rate in the dark than in the light (Fig. 6 and Table S3). Indeed, dark adsorption is at the lower limits of the parameter constraint range of the MCMC procedure, suggesting that P-HM2 may have effectively zero adsorption in the dark. In contrast, model estimates cannot reject the hypothesis that adsorption was effectively constant for P-SSP7. The convergence of MCMC chains further supports the robustness of the model-based inferences (Table S4 and Fig. S1b and S1c). Notably, other candidate models with intracellular mechanisms that delay lysis can also reproduce similar population-level features (Text S1 and Fig. S5).

10.1128/mSystems.00586-19.5 FIG S5 *In silico* experiments with candidate models that reproduce a slowdown of the population lysis. The initial model is represented by a dashed black line, and the candidate models are in solid colored lines. Download FIG S5, PDF file, 1.6 MB. Copyright © 2020 Demory et al. 2020 Demory et al. This content is distributed under the terms of the Creative Commons Attribution 4.0 International license.

10.1128/mSystems.00586-19.6 TEXT S1 Candidate models. Download Text S1, PDF file, 0.1 MB. Copyright © 2020 Demory et al. 2020 Demory et al. This content is distributed under the terms of the Creative Commons Attribution 4.0 International license.

## DISCUSSION

We investigated the impact of light and dark conditions on the infection of *Prochlorococcus* by cyanophage using a combination of experiments, nonlinear population models, and model-data integration. We found that light-dependent differences in viral adsorption to hosts help explain population-level changes in both virus and host abundances given growth under diurnal conditions. These light-dependent differences are strain specific. Estimated adsorption rates vary markedly during the light versus the dark for P-HM2 but not for P-SSP7. This suggests that viruses, in addition to hosts, may have light-dependent differences in their life history traits at the cellular scale that impact dynamics at population scales.

In our model-fitting procedure, we evaluated the possibility that light could affect adsorption, latent period, and burst size. We only found evidence for light-dependent variation in adsorption rate for the phage P-HM2. In contrast, P-SSP7 dynamics were explained by light-driven variation in the host growth rate only. Our results for both viruses corroborate the observations in reference 37, supporting evidence of mechanisms of light-driven *Prochlorococcus* infection dynamics for P-HM2 but not for P-SSP7. However, model estimations for P-SSP7 burst size were higher than the values reported in the literature, suggesting that other factors may be involved in the dynamics. The imputed failure of P-HM2 to adsorb to MED4 in the dark indicates that adsorption could be directly modulated by light (36). Light-dependent variation in adsorption has also been reported in cyanophage infecting *Synechococcus* (36, 39) and in coccolithoviruses (47).

There are multiple reasons why P-HM2 may have evolved light-dependent viral LHTs. First, exposure to UV is a critical factor degrading viral particles outside the host cell (19). During the night, there is both less UV and (potentially) elevated predation rates of cyanobacteria by eukaryotic grazers (52, 53). Therefore, remaining outside the host cell during the night could effectively amount to a survival strategy by avoiding predation by grazers on the viral host (20). The evolution of light-dependent LHTs may also be host dependent, e.g., as a response to variation in host availability.

Despite our focus on light-driven traits, our approach revealed other mechanisms driving variation in host-virus population dynamics. The failure of a light-driven virus-host population model to recapitulate the persistence of host cells suggests that other feedback mechanisms may limit host mortality, even when virus densities are relatively high. Using a variant of the original model, we found evidence consistent with lysis inhibition at high viral densities (54). Mechanisms consistent with lysis inhibition include decreases in viral infectivity, an increase in the production of defective viral particles, or slowdowns in host physiology. Such slowdowns reflect the potential reciprocal influence of processes at cellular and population scales. The relevance of such slowdowns will vary with the environment. For example, in marine surface environments, cyanophage densities do not typically exceed 10^6^ ml^−1^, and so it remains unclear whether the candidate feedback mechanism is an adaptive response to the high density of infected hosts or arises incidentally given ecological conditions outside typically encountered ranges. Further work is necessary to disentangle process from pattern.

In closing, we found that light-dependent viral life history traits can substantively change the dynamics of *Prochlorococcus* and cyanophage. This finding reinforces and extends the consequences of prior results showing that viral traits differ between light and dark, albeit under fixed conditions. In the marine environment, adaptation to light has been shown to drive differences in physiology among *Prochlorococcus* cyanobacteria, as well as evolutionary adaptation between light-associated ecotypes. Our study suggests that exploring variation in virus-associated light-dependent life history traits may also reveal ways in which viruses partition their environment, both in terms of host specificity and via differential infection of hosts over light-dark cycles.

## MATERIALS AND METHODS

### Experimental design and data attribution.

The experimental data analyzed here comprise data from two published sources, Liu et al. (45) and Liu et al. (37), and new data collected to link infection-level dynamics with population-level dynamics. Specifically, the host growth data in Fig. 2 were previously reported in Liu et al. (45). The infection data in Fig. 1C and D, as well as the host and phage abundances before 60 h in Fig. 4 and 5, are reported in Liu et al. (37). The host and phage abundance data after 60 h in Fig. 4 and 5, as well as the viral decay data reported in Fig. S7 in the supplemental material, are newly reported here. Details of the experimental procedures are described in the following sections, with full quotations used when methods are equivalent to those reported in reference 37. We include full method descriptions for completeness.

### Culture conditions.

Culture conditions were as described by R. Liu et al. (37):

Axenic *Prochlorococcus* strains were grown in Port Shelter (Hong Kong) seawater-based Pro99 medium (55). Batch cultures were incubated at 23°C in continuous light (25 μmol quanta m^−2^ s^−1^) or a 14h light:10h dark cycle (35 μmol quanta m^−2^ s^−1^ in the light period). Cultures were acclimated in the same condition for at least three months before they were used for the experiments.

### Infection of synchronized *Prochlorococcus* cells under light-dark cycles.

Infection of *Prochlorococcus* cells under light-dark cycles was as described by R. Liu et al. (37):

*Prochlorococcus* cells were acclimated under light-dark cycles for at least three months and were synchronized, as determined by flow cytometry. Mid-log cells were infected at different times of a light-dark cycle at a phage/host ratio of 0.02. Times of infection were 0.5, 6, 12 hours. Each experiment was replicated at least two times.

### Cyanophage decay rates under light or dark.

To measure the decay rates, fresh lysates of cyanophages P-HM2 and P-SSP7 were prepared by adding 300 μl virus stocks into 30 ml mid-log *Prochlorococcus* MED4 culture. After the infected culture became clear, cell debris was removed using a 0.2-μm polycarbonate filter and the supernatant containing phage particles was stored at 4°C in the dark. During the viral decay experiment, aliquots of viral lysates were incubated at 23°C at a light intensity of 27 μmol photons m^−2^ s^−1^, and aliquots were incubated at the same temperature in the dark (56). Samples were taken from each tube every 2 days over 10 days. To measure the loss of viral infectivity, the numbers of PFU were measured (57). Briefly, 500 μl serially diluted viral lysate was added to 2 ml *Prochlorococcus* MED4 (grown to mid-log phase in Pro99) in glass tubes and incubated at room temperature for 15 min to allow phage adsorption. Incubated cultures were then combined with UltraPure low-melting-point agarose (Invitrogen) at a final concentration of 0.5%. The EZ55 *Alteromonas* helper bacterium (58) was added to every plate. Plaques began to appear 7 days later on plates that were incubated at 23°C at a light intensity of 19 μmol photons m^−2^ s^−1^. Each sample was measured with three technical replicates.

### Flow cytometry and cell cycle analysis.

Flow cytometry and cell cycle analysis were performed as described by R. Liu et al. (37):

*Prochlorococcus* cells were preserved by mixing 100 μL culture with 2 μL 50% glutaraldehyde to a final concentration of 1% and were stored at −80°C. Cells were enumerated by a flow cytometer (BD FACSCalibur) with the CellQuestPro software. We followed a published protocol to determine the percentage of cells in each cell cycle stage (22). Briefly, *Prochlorococcus* cells were stained with the DNA stain SYBR Green (Invitrogen) and flow cytometry data were analyzed with the ModfitLT software.

### Quantification of cyanophage.

Cyanophage were quantified as described by R. Liu et al. (37):

Total phage particles were collected on a 0.02 μm Whatman Anodisc filter, stained with SYBR gold (Molecular Probes), and counted under an epifluorescence microscope (59, 60). At least five discrete fields on a filter were photographed using the SPOT Advanced Imaging software and fluorescent dots representing phage particles were counted manually.

During infection, extracellular phage DNA was quantified using a quantitative polymerase chain reaction (qPCR) method (61). Briefly, infected *Prochlorococcus* cultures were filtered through 0.2 μm polycarbonate filters in a 96-well filter plate (Pall). Filtrates containing extracellular phage particles were diluted 100-fold in dH_2_O and were then used as templates for qPCR reactions in a 384-well plate. A qPCR reaction contained 4.6 *μ*L template, 0.2 *μ*L forward primer (10 *μ*M), 0.2 *μ*L reverse primer (10 *μ*M), and 5 *μ*L iTaq Universal SYBR Green Supermix. The LightCycler 480 Real-Time PCR System (Roche Diagnostics) was used for thermal cycling, which consisted of an initial activation step of 5 min at 95°C, 45 amplification cycles of 10 s at 95°C and 60 s at 60°C, and a melting curve analysis at the end. The number of cyanophage in each well was quantified using a standard curve generated from phage particles that were enumerated by epifluorescence microscopy.

Measurement of phage DNA copies by qPCR provides an ∼1:1 relationship with phage particle counts (37, 51).

### Model simulations.

Model analyses were performed with MATLAB version 9.2.0 (The MathWorks, Inc., 2017; Natick, MA). Infection dynamics were simulating using MATLAB ODE solver *ode*45 (62) (The MathWorks, Inc.), which uses a higher-order Runge-Kutta method (63).

### Estimation of the best parameter sets. (i) General procedure.

We aimed to estimate the parameter set θ which best described the measurements of host and virus abundances, given a particular model. The procedure consisted of two steps. First, we estimated host growth parameters *θ*_host_ for the model without viruses (equation 1). Next, we used *θ*_host_ in the model with viruses (equation 5 and 6) and estimated the infection parameters θ_infection_ for each hypothesis. We estimated parameters by minimizing an objective function that describes the error between model fit and experimental data.

### (ii) The objective function.

The objective function calculated error between the model fits and the measurements as follows:

(7) ϵ(θ)=∑z[1nhostlog⁡(y^t,hostyt,host)2+1nviruslog⁡(y^t,virusyt,virus)2]

where ϵ is the total error for the parameter set *θ*, over *z* experiments. We decomposed the error into host and virus, with *y_t_*_,host_ denoting measurements and y^t,host denoting model fits at time *t*. For the host error, y^t,host=N(t), where *N*(*t*) is the sum of the susceptible, exposed, and infected host cell estimations. Then, the total error was the sum of the host and virus errors for the whole set of experiments.

### (iii) Algorithms.

We first sampled the parameter space with Latin hypercube sampling (LHS) (64) for 20,000 parameter sets for each hypothesis and model. We calculated an initial error for each parameter set by running the dynamical model and calculating the objective function (equation 7). Next, we implemented a Markov chain Monte Carlo (MCMC) procedure for the 10 best initial parameter sets. We used two “burn-in” periods (running periods that allow the convergence of the chains). The 10 parameter distributions from the resulting chains each consisted of 5,000 parameter sets. The distributions tended to overlap; when this happened, we chose the best distribution from the overlapping set, as quantified by the median of the error (equation 7). We used the MCMC toolbox for MATLAB, implementing the DRAM algorithm (65).

### (iv) Estimation of the host growth parameters θ_host_.

For the host growth parameter sets θ_host_ = (*α*, *L*_opt_, *μ*_max_, *K_L_*, *ω*, *K*), we used the procedure described above to estimate parameters for *Prochlorococcus* strain MED4. Parameters of the growth-irradiance curves (α, *L*_opt_, and μ_max_; equation 4) were constrained by the data from Moore and Chisholm (50), whereas *K_L_* and ω were not constrained. The carrying capacity *K* was fixed and considered a constant (*K* = 3.10^9^ cell ml^−1^ [according to reference 66] for nonaxenic cultures).

### (v) Estimation of the infection parameters θ_infection_.

To estimate the parameter set θ_infection_ = (ϕ, λ, β), we fixed the host growth parameters estimated previously and estimated the infection parameters relative to the hypotheses *H*0 to *H*7. Depending on the hypothesis, the estimated parameter could be constant during the experiments (no relation with light or dark condition) or a piecewise function (with differing light and dark values). The estimated parameters were the adsorption rate ϕ, the latent period λ, and the burst size β. Viral decay rates were estimated experimentally as the slope of log(viral concentration) regression under light or dark conditions and fixed (Fig. S2).

### Quantifying the best model hypothesis.

To quantify the best model under hypotheses *H*0 to *H*7, we computed an Akaike information criterion (AIC) and a Bayesian information criterion (BIC) (67) for virus and host (when data were available) according to the following equations (equations 8 and 9):

(8) $${\text{AIC}}_{j}=2k_{j}+\sum_{z}\left(\epsilon_{\text{host}}+\epsilon_{\text{virus}}\right)$$

(9) $${\text{BIC}}_{j}=k_{j}\;\log(n_{\text{virus}}+n_{\text{host}})+\underset{z}{\sum }\left(\epsilon_{\text{host}}+\epsilon_{\text{virus}}\right)$$

with

ϵhost=nhost log⁡(∑t(yhost−y^host)2nhost)

and

ϵvirus=nvirus log⁡(∑t(yvirus−y^virus)2nvirus)

These criteria are computed depending on the hypothesis *j* with the number of parameters to be estimated *k_j_* (3 parameters for *H*0, 4 for hypotheses *H*1, *H*2, and *H*3, 5 for hypotheses *H*4, *H*5, and *H*6, and 6 for hypothesis *H*7) (Fig. 2b), *n*_host_ and *n*_virus_ being the total number of data points for host and virus, respectively, *z* being the treatment, *y*_host_ and *y*_virus_ being the data points for the hypothesis *j* and the treatment *z* at time point *t* for host and virus, respectively, and y^host and y^virus being the model estimation for the hypothesis *j* and the treatment *z* at time point *t* for host and virus, respectively.

### Estimation of adsorption with experimental data.

We estimated the experimental adsorption from the viral data (*V*) of Fig. 1. We assumed that the host growth was negligible in the first hours of the experiments (*t* < 6 h) and estimated the adsorption as follows:

(10) $$\phi_{\text{estimation}}=\frac{r}{P_{\left(t=0\right)}}$$

where *r* is the slope of −log⁡V versus time and $P_{\left(t=0\right)}$ is the initial concentration of *Prochlorococcus* at time *t* = 0.

### Data availability statement.

All data are available for use and reuse. The full data set and code are available at https://doi.org/10.5281/zenodo.3308790. As noted in “Experimental design and data attribution” above, the analysis here includes data from both published and unpublished sources. Data in Fig. 1c and d are from reference 37. Data in Fig. 2a are from reference 45. Data in Fig. 4 and 5 until 60 h are reused from reference 37, and new data are used for time points after 60 h. Data for Fig. S2 are original.
