# Correction for Demory et al., “Linking Light-Dependent Life History Traits with Population Dynamics for *Prochlorococcus* and Cyanophage”

**DOI:** 10.1128/mSystems.01005-21

**Published:** 2021-09-14

**Authors:** David Demory, Riyue Liu, Yue Chen, Fangxin Zhao, Ashley R. Coenen, Qinglu Zeng, Joshua S. Weitz

**Affiliations:** a School of Biological Sciences, Georgia Institute of Technology, Athens, Georgia, USA; b Division of Life Science, The Hong Kong University of Science and Technology, Hong Kong, China; c Department of Ocean Science, The Hong Kong University of Science and Technology, Hong Kong, China; d School of Physics, Georgia Institute of Technology, Atlanta, Georgia, USA; e HKUST Shenzhen Research Institute, Shenzhen, China; f Hong Kong Branch of Southern Marine Science and Engineering, Guangdong Laboratory (Guangzhou), HKUST, Hong Kong, China

## AUTHOR CORRECTION

Volume 5, no. 2, e00586-19, 2020, https://doi.org/10.1128/mSystems.00586-19.

The original publication of this paper erroneously included a factor of 2 in the denominator rather than the numerator when representing the state transitions corresponding to intracellular transitions from infection to lysis in the nonlinear population model. As a result, the estimated latent periods were underreported by a factor of 4. The corrected latent period ($\lambda_{\text{corrected}}$) should be $\lambda_{\text{corrected}}=4\lambda_{\text{original}}$. This rescaling does not impact the MCMC procedure, model-data fits, or conclusions. Instead, we find that corrected latent periods are in the range of 14.4 to 15.6 h for phage P-HM2 (rather than 3.6 to 3.9 h, originally) and 9.6 to 10.4 h for phage P-SSP7 (rather than 2.4 to 2.6 h, originally); these corrected values are in fact in closer concordance with estimated average latent periods of ∼8 h measured in experiments for phage P-SSP7 (e.g., D. Lindell, J. D. Jaffe, M. L. Coleman, M. E. Futschik, et al., Nature 449:83–86, 2007, https://doi.org/10.1038/nature06130).

Page 4: Equation 5 should read as follows.

$$\frac{\mathrm{dS}}{\mathrm{dt}}=\mu S\left(1-\frac{N}{K}\right)-\omega S-\phi \mathrm{SV}$$

$$\frac{\mathrm{dE}}{\mathrm{dt}}=\phi \mathrm{SV}-\omega E-\frac{2}{\lambda }E$$

$$\frac{\mathrm{dI}}{\mathrm{dt}}=\frac{2}{\lambda }E-\omega I-\frac{2}{\lambda }I$$

$$\frac{\mathrm{dV}}{\mathrm{dt}}=\frac{2}{\lambda }\beta I-\phi \mathrm{NV}-\delta V$$

Page 6: Figure 3 should appear as shown below, with 1/(2λ) changed to 2/λ.

**FIG 3:**
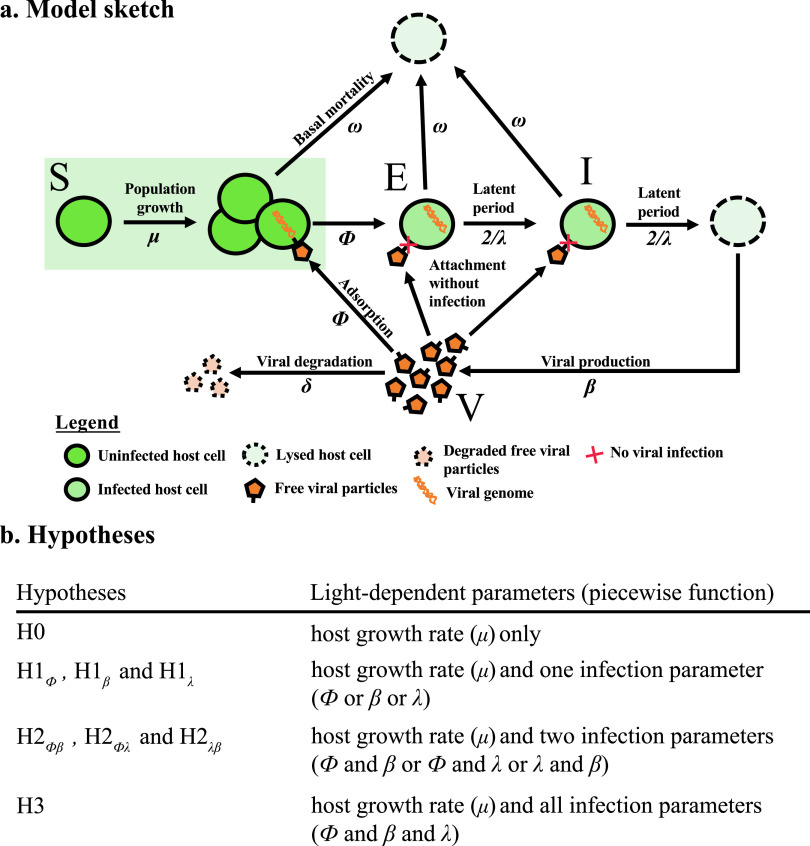
Model infection parameter distributions.

Page 7: Equation 6 should read as follows.

$$\frac{\mathrm{dS}}{\mathrm{dt}}=\mu S\left(1-\frac{N}{K}\right)-\omega S-\phi \mathrm{SV}$$

$$\frac{\mathrm{dE}}{\mathrm{dt}}=\phi \mathrm{SV}-\omega E-\frac{2}{\lambda }E+\phi \mathrm{IV}$$

$$\frac{\mathrm{dI}}{\mathrm{dt}}=\frac{2}{\lambda }E-\omega I-\frac{2}{\lambda }I-\phi \mathrm{IV}$$

$$\frac{\mathrm{dV}}{\mathrm{dt}}=\frac{2}{\lambda }\beta I-\phi \mathrm{NV}-\delta V$$

Page 7, line 5: The equation should read as follows.

ϕV/[2/λ + ω + ϕV]

Page 9: Figure 6 should appear as shown below, with the latent period values (λ) multiplied by a factor of 4 (middle panels).

**FIG 6:**
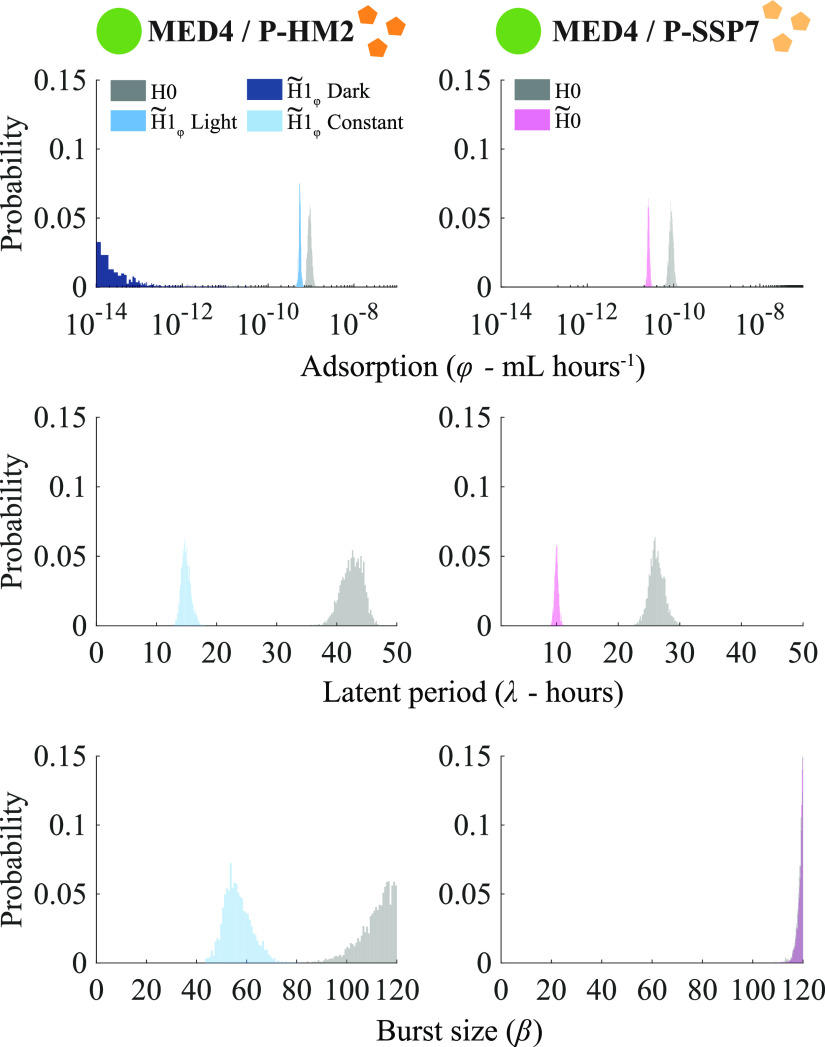
Description of the model.

Supplemental material: In Fig. S1, the latent period chains (λ) were multiplied by a factor of 4 (panels b and c). In Table S2, the unit of the latent period (λ) is shown in hours and not per hours. In Table S4, the latent period values (λ) were multiplied by a factor of 4 for the two pairs HM2/MED4 and SSP7/MED4. Revised supplemental material is posted online.

