# Supplementary figures and images for: Linking Light-Dependent Life History Traits with Population Dynamics for Prochlorococcus and Cyanophage
Source: mSystems. 2020 Mar 31;5(2):e00586-19. doi: 10.1128/mSystems.00586-19 (PMC7112961; doi:10.1128/mSystems.00586-19)

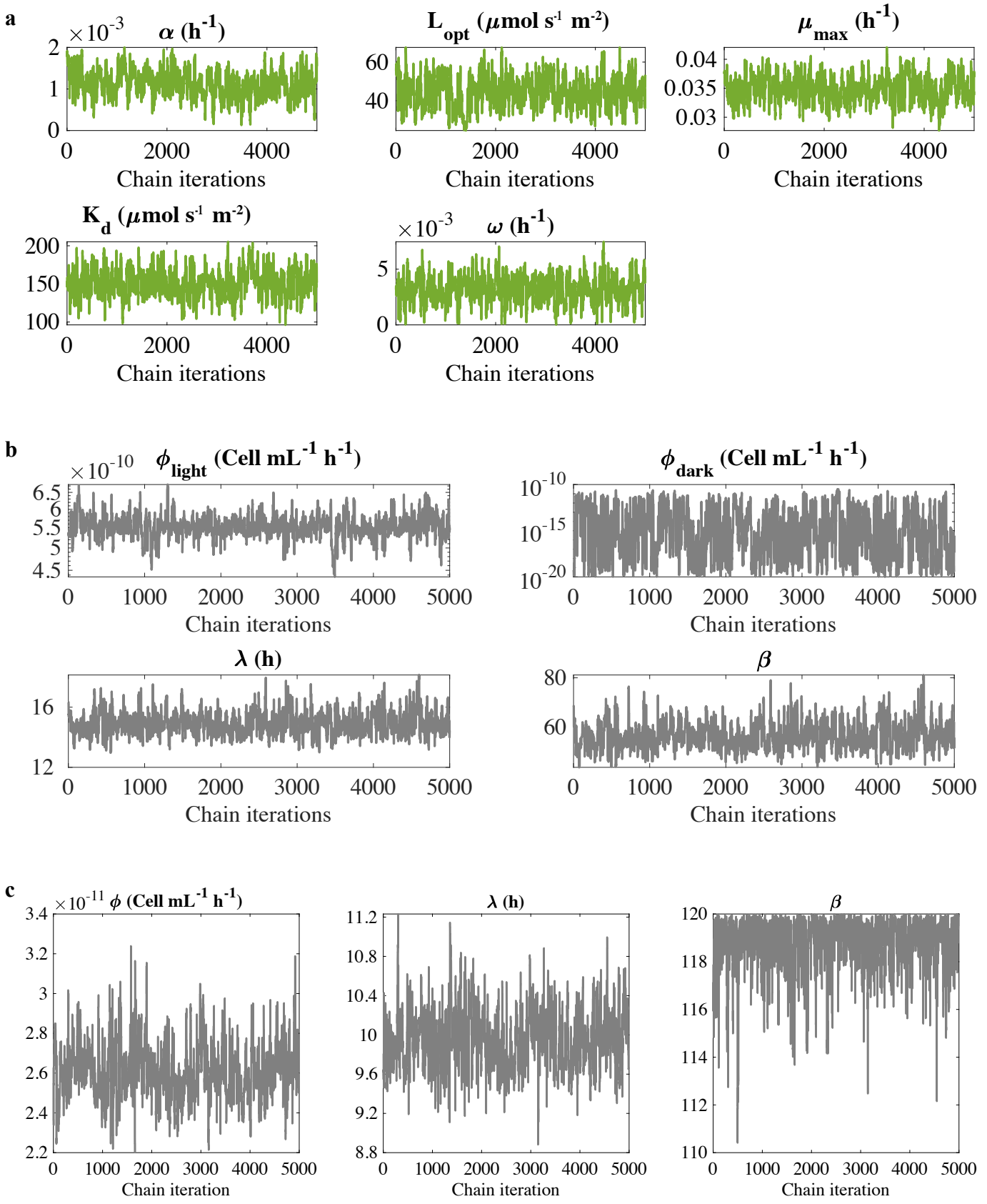

Supplement: FIG S1 [file msystems.00586-19-sf001_revised.pdf]

**a**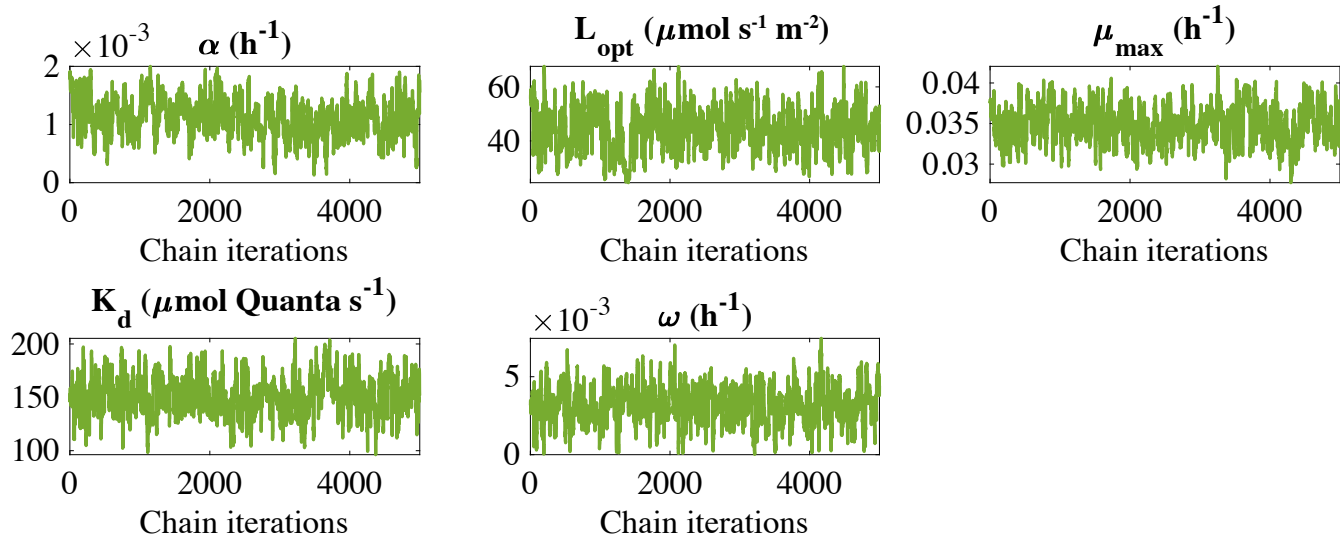**b**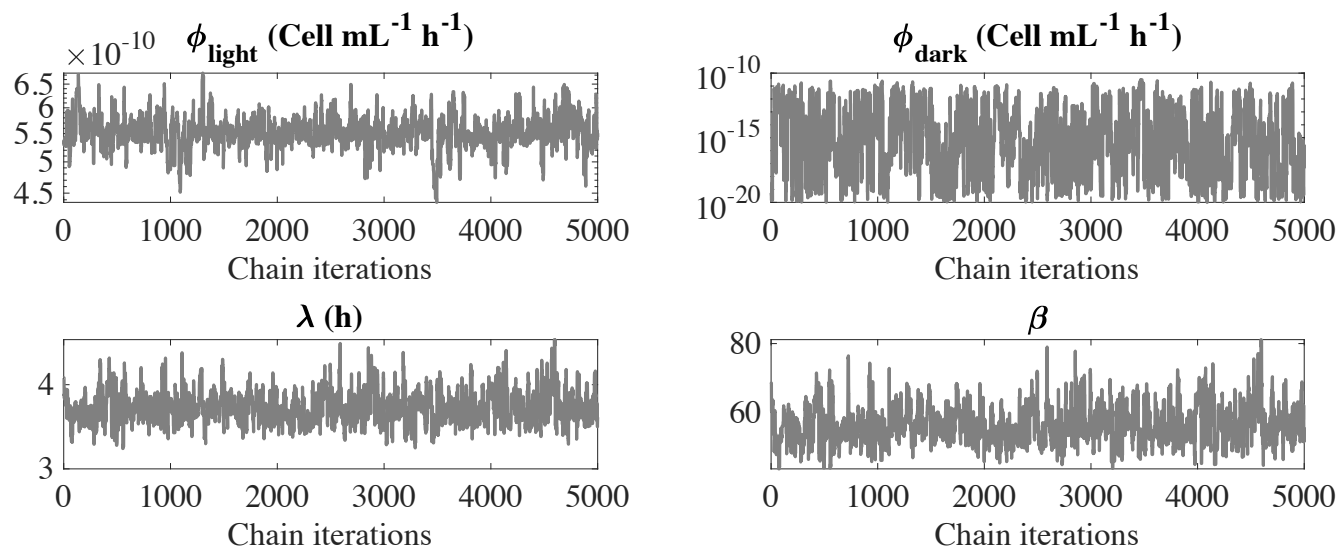**c**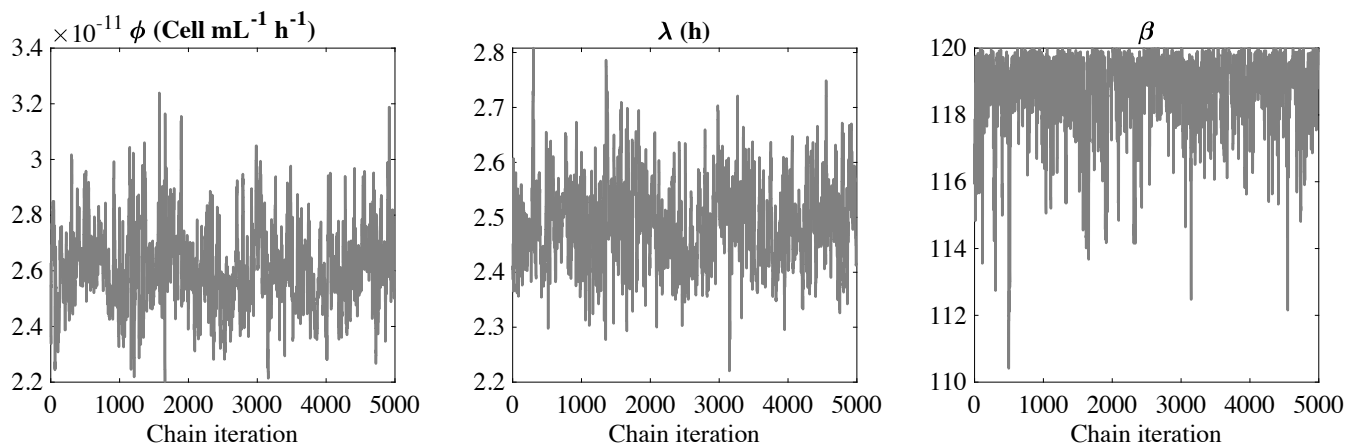

Supplement: FIG S1 [file msystems.00586-19-sf001_original.pdf]

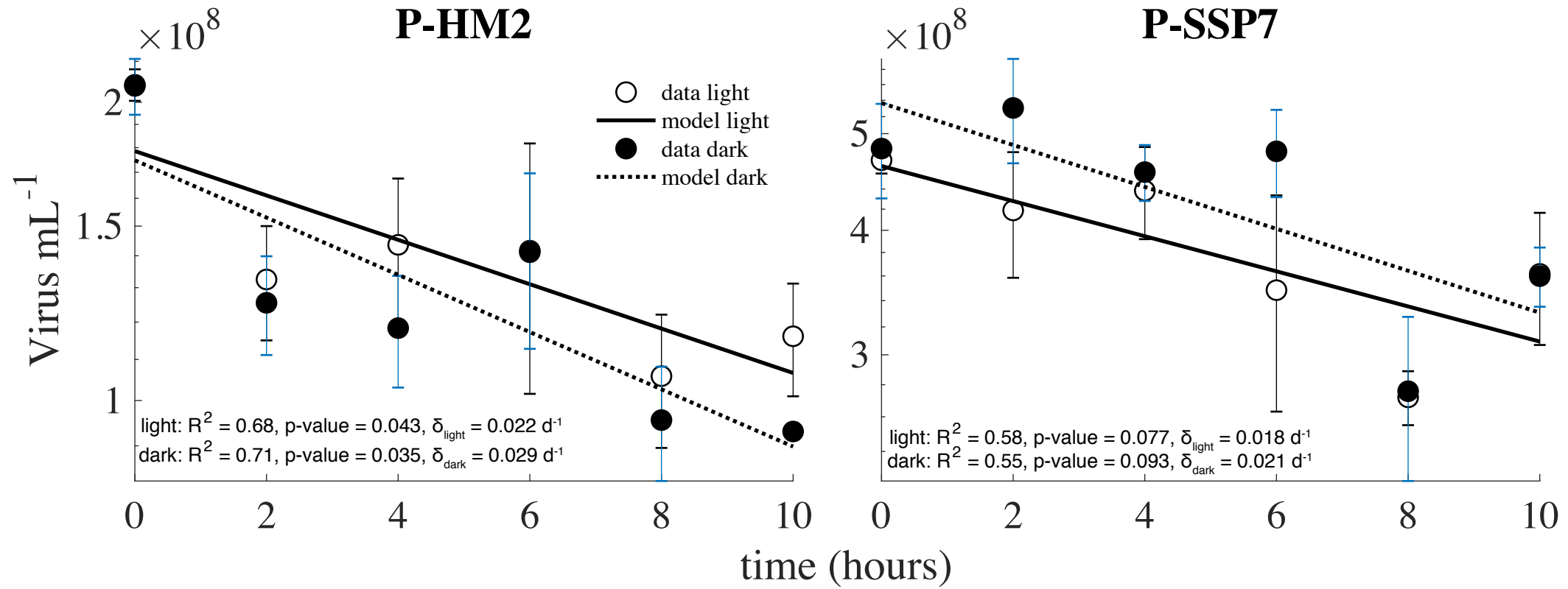

Supplement: FIG S2 [file msystems.00586-19-sf002.pdf]

**a**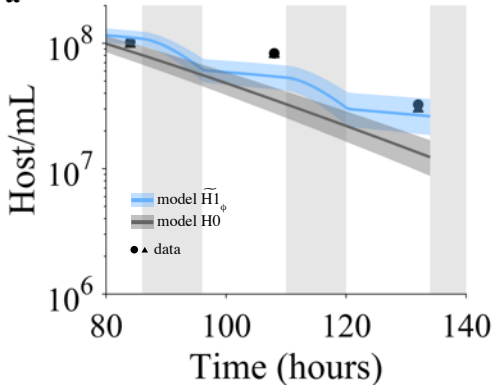**b**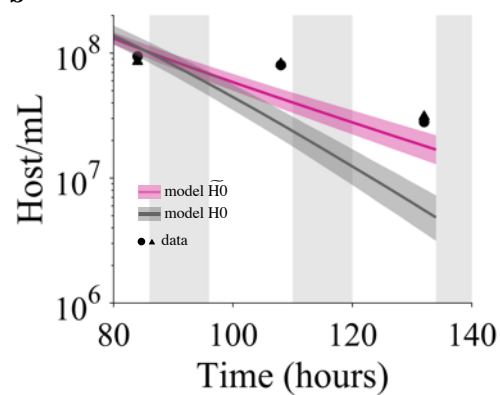**c**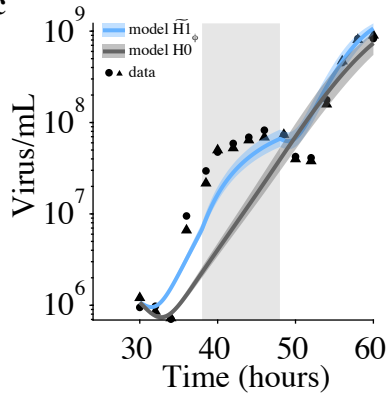

Supplement: FIG S4 [file msystems.00586-19-sf004.pdf]

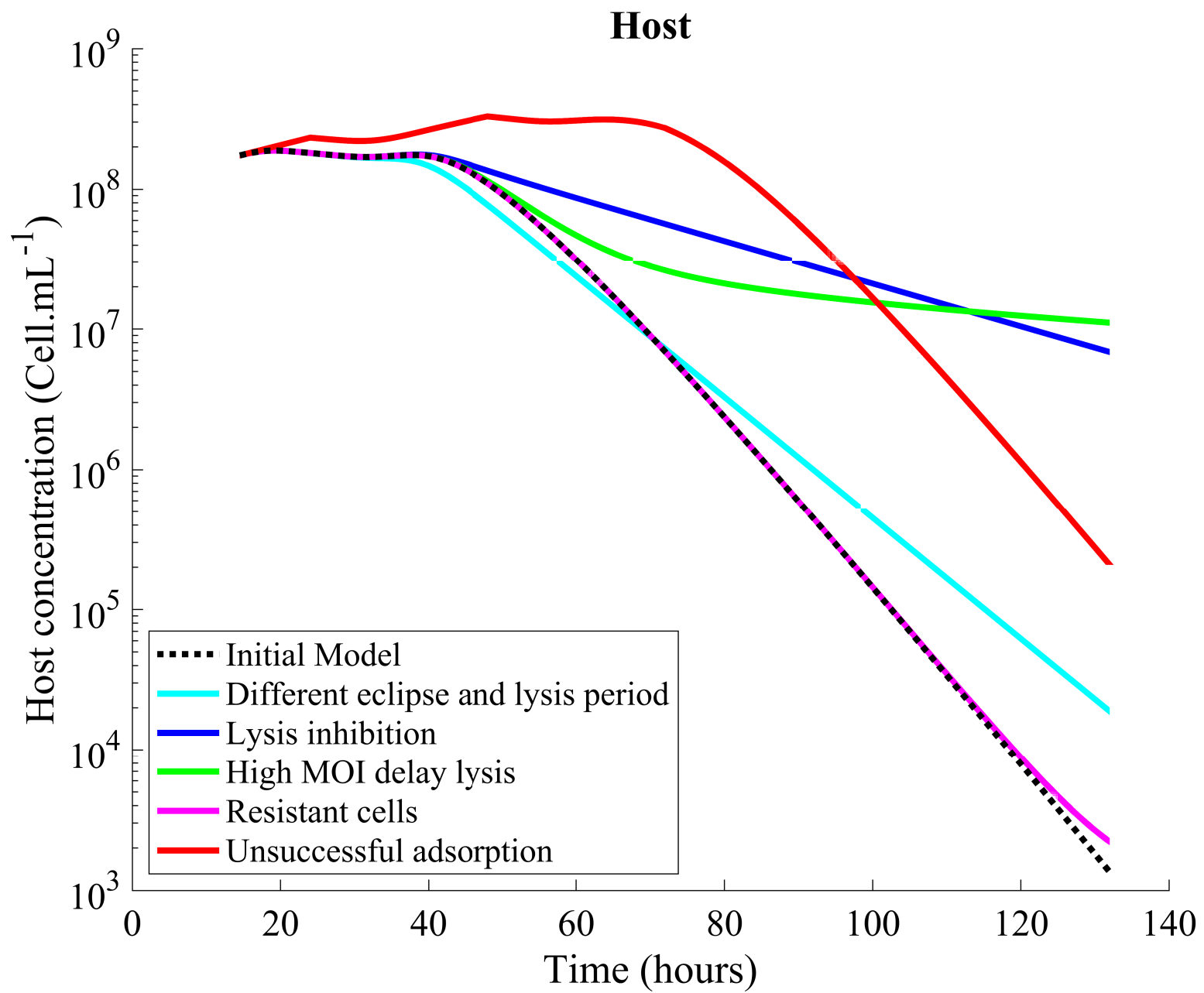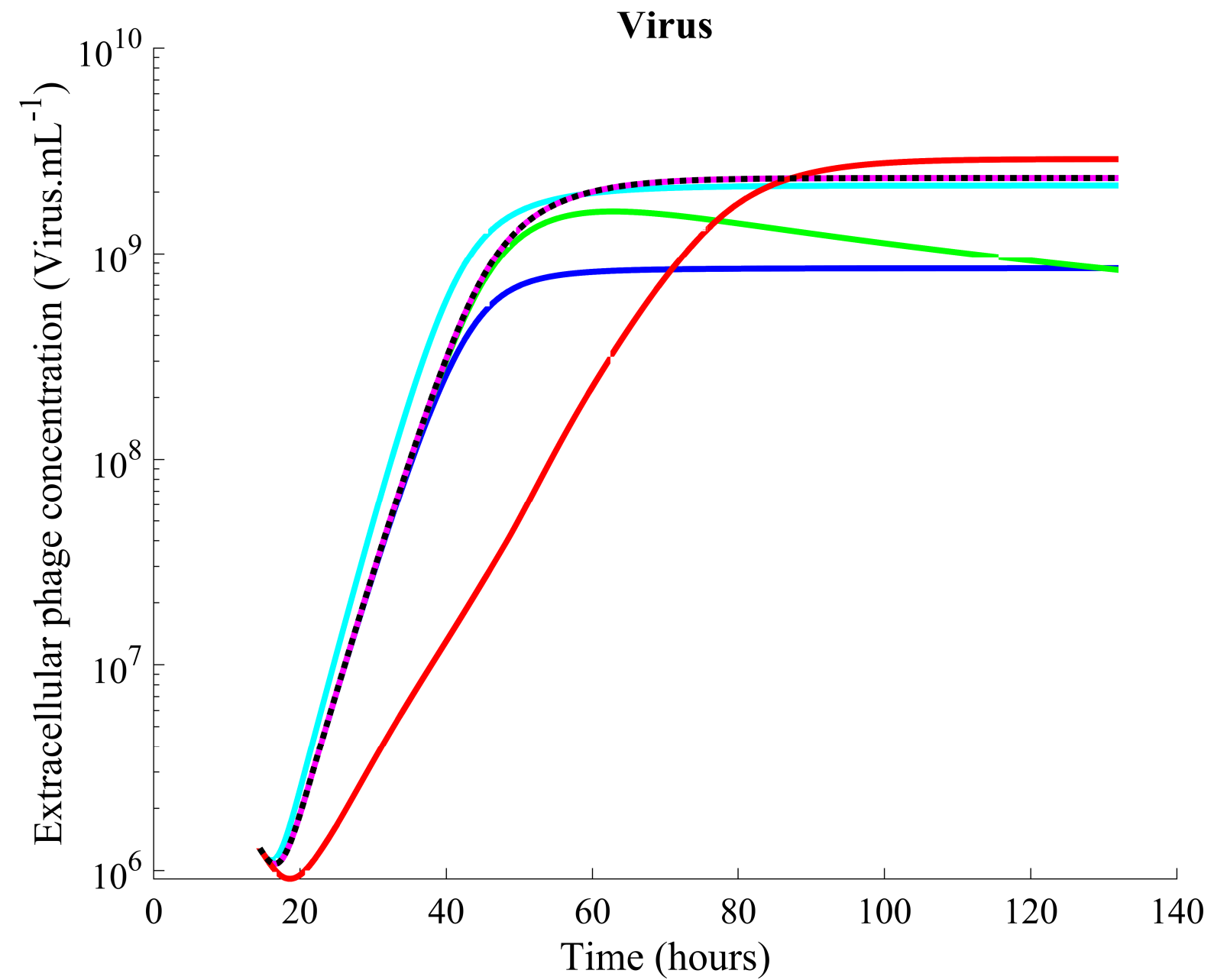

Supplement: FIG S5 [file msystems.00586-19-sf005.pdf]
