# Supplementary material for: Linking Light-Dependent Life History Traits with Population Dynamics for Prochlorococcus and Cyanophage
Source: mSystems. 2020 Mar 31;5(2):e00586-19. doi: 10.1128/mSystems.00586-19 (PMC7112961; doi:10.1128/mSystems.00586-19)
Supplement: TABLE S1 [file msystems.00586-19-st001.pdf]

Parameters	Model	Measurements from [50]
α	$1.10\ 10^{-3} \pm 3.10\ 10^{-4}$	$5.83\ 10^{-4} \pm 4.17\ 10^{-5}$
L_{opt}	44.78 ± 7.98	45 ± 7
μ_{max}	0.035 ± 0.0021	0.0262 ± 0.0025
K_L	151.59 ± 17.51	–
ω	0.0032 ± 0.0012	–
