# Supplementary material for: Linking Light-Dependent Life History Traits with Population Dynamics for Prochlorococcus and Cyanophage
Source: mSystems. 2020 Mar 31;5(2):e00586-19. doi: 10.1128/mSystems.00586-19 (PMC7112961; doi:10.1128/mSystems.00586-19)
Supplement: TABLE S2 [file msystems.00586-19-st002_original.pdf]

Parameters	Meaning	unit
μ	Host growth rate	h^{-1}
K	Host carrying capacity	Cell ml^{-1}
ω	Host mortality	h^{-1}
ϕ	Viral adsorption	ml h^{-1}
λ	Viral lysis	h^{-1}
β	Burst size	no dimension
δ	Viral decay	h^{-1}
