# Supplementary material for: Linking Light-Dependent Life History Traits with Population Dynamics for Prochlorococcus and Cyanophage
Source: mSystems. 2020 Mar 31;5(2):e00586-19. doi: 10.1128/mSystems.00586-19 (PMC7112961; doi:10.1128/mSystems.00586-19)
Supplement: FIG S3 [file msystems.00586-19-sf003.pdf]

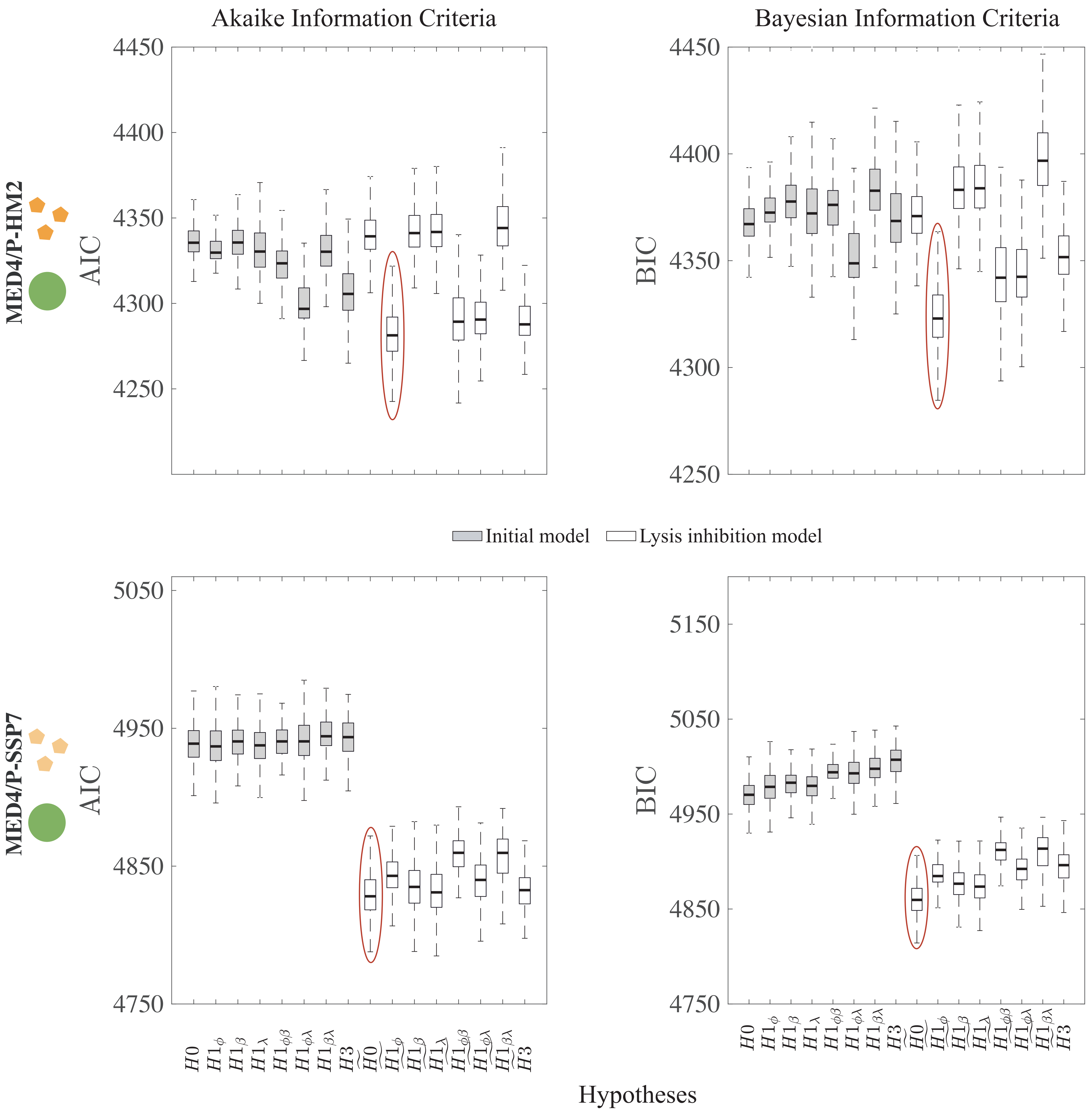
Akaike Information Criteria
Bayesian Information Criteria
MED4/P-HM2
MED4/P-SSP7
AIC
BIC
4450
4400
4350
4300
4250
5050
4950
4850
4750
5150
Initial model
Lysis inhibition model
$H0$
$H1_\phi$
$H1_\beta$
$H1_\lambda$
$H1_{\phi\beta}$
$H1_{\phi\lambda}$
$H1_{\beta\lambda}$
$H3$
$\widetilde{H0}$
$\widetilde{H1_\phi}$
$\widetilde{H1_\beta}$
$\widetilde{H1_\lambda}$
$\widetilde{H1_{\phi\beta}}$
$\widetilde{H1_{\phi\lambda}}$
$\widetilde{H1_{\beta\lambda}}$
$\widetilde{H3}$
Hypotheses
