# Supplementary material for: Linking Light-Dependent Life History Traits with Population Dynamics for Prochlorococcus and Cyanophage
Source: mSystems. 2020 Mar 31;5(2):e00586-19. doi: 10.1128/mSystems.00586-19 (PMC7112961; doi:10.1128/mSystems.00586-19)
Supplement: TABLE S3 [file msystems.00586-19-st003.pdf]

P-HM2

Parameters	From this study	From [51]
Adsorption	9.80 10^{-12} ml h^{-1} (Dark) and 2.55 10^{-10} ml h^{-1} (Light)	–
Latent period	≤ 6 h	5 (5 – 6.5) h
Burst size	–	28 (18 – 30) Virus $cell^{-1}$

P-SSP7

Parameters	From this study	From [51]
Adsorption	8.39 10^{-11} ml h^{-1} (Dark) and 7.98 10^{-11} ml h^{-1} (Light)	–
Latent period	≤ 6 h	5 (5 – 9) h
Burst size	–	40 (37 – 74) Virus $cell^{-1}$
