# Supplementary material for: Linking Light-Dependent Life History Traits with Population Dynamics for Prochlorococcus and Cyanophage
Source: mSystems. 2020 Mar 31;5(2):e00586-19. doi: 10.1128/mSystems.00586-19 (PMC7112961; doi:10.1128/mSystems.00586-19)
Supplement: TABLE S4 [file msystems.00586-19-st004_revised.pdf]

HM2/MED4

	$H0$	$H5_{\phi\lambda}$	$\widetilde{H}1_{\phi}$
Adsorption	$9.3\ 10^{-10}$ ($8.7\ 10^{-10}$ – $9.8\ 10^{-10}$)	–	–
Adsorption light	–	$1.6\ 10^{-9}$ ($1.5\ 10^{-9}$ – $1.7\ 10^{-9}$)	$5.5\ 10^{-10}$ ($5.4\ 10^{-10}$ – $5.7\ 10^{-10}$)
Adsorption dark	–	$4.5\ 10^{-12}$ ($1.3\ 10^{-12}$ – $1.2\ 10^{-11}$)	$3.1\ 10^{-16}$ ($3.0\ 10^{-18}$ – $4.7\ 10^{-14}$)
Latent period	42.4 (41.2 – 43.6)	–	14.8 (14.4 – 15.6)
Latent period light	–	32 (31.2 – 33.2)	–
Latent period dark	–	56 (51.6 – 62)	–
Burst size	113.0 (107.2 – 117.1)	116.4 (111.6 – 118.5)	56.1 (52.8 – 59.6)

SSP7/MED4

	$H0$	$H2_{\phi}$	$\widetilde{H}0$
Adsorption	$8.8\ 10^{-11}$ ($8.2\ 10^{-11}$ – $9.6\ 10^{-11}$)	–	$2.6\ 10^{-11}$ ($2.5\ 10^{-11}$ – $2.7\ 10^{-11}$)
Adsorption light	–	$8.7\ 10^{-11}$ ($7.4\ 10^{-11}$ – $10.0\ 10^{-11}$)	–
Adsorption dark	–	$9.4\ 10^{-11}$ ($7.8\ 10^{-11}$ – $1.1\ 10^{-10}$)	–
Latent period	26.4 (25.6 – 26.8)	26.4 (25.6 – 27.2)	10 (9.6 – 10.4)
Burst size	119.1 (118.0 – 119.6)	119.1 (118.2 – 119.6)	119.1 (118.2 – 119.6)
