# Supplementary material for: Linking Light-Dependent Life History Traits with Population Dynamics for Prochlorococcus and Cyanophage
Source: mSystems. 2020 Mar 31;5(2):e00586-19. doi: 10.1128/mSystems.00586-19 (PMC7112961; doi:10.1128/mSystems.00586-19)
Supplement: TABLE S4 [file msystems.00586-19-st004_original.pdf]

**HM2/MED4**

| | $H0$ | $H5_{\phi\lambda}$ | $\widetilde{H1_{\phi}}$ |
|---|---|---|---|
| Adsorption | $9.3\ 10^{-10}$ ($8.7\ 10^{-10}$ – $9.8\ 10^{-10}$) | – | – |
| Adsorption light | – | $1.6\ 10^{-9}$ ($1.5\ 10^{-9}$ – $1.7\ 10^{-9}$) | $5.5\ 10^{-10}$ ($5.4\ 10^{-10}$ – $5.7\ 10^{-10}$) |
| Adsorption dark | – | $4.5\ 10^{-12}$ ($1.3\ 10^{-12}$ – $1.2\ 10^{-11}$) | $3.1\ 10^{-16}$ ($3.0\ 10^{-18}$ – $4.7\ 10^{-14}$) |
| Latent period | 10.6 (10.3 – 10.9) | – | 3.7 (3.6 – 3.9) |
| Latent period light | – | 8.0 (7.8 – 8.3) | – |
| Latent period dark | – | 14.0 (12.9 – 15.5) | – |
| Burst size | 113.0 (107.2 – 117.1) | 116.4 (111.6 – 118.5) | 56.1 (52.8 – 59.6) |

**SSP7/MED4**

| | $H0$ | $H2_{\phi}$ | $\widetilde{H0}$ |
|---|---|---|---|
| Adsorption | $8.8\ 10^{-11}$ ($8.2\ 10^{-11}$ – $9.6\ 10^{-11}$) | – | $2.6\ 10^{-11}$ ($2.5\ 10^{-11}$ – $2.7\ 10^{-11}$) |
| Adsorption light | – | $8.7\ 10^{-11}$ ($7.4\ 10^{-11}$ – $10.0\ 10^{-11}$) | – |
| Adsorption dark | – | $9.4\ 10^{-11}$ ($7.8\ 10^{-11}$ – $1.1\ 10^{-10}$) | – |
| Latent period | 6.6 (6.4 – 6.7) | 6.6 (6.4 – 6.8) | 2.5 (2.4 – 2.6) |
| Burst size | 119.1 (118.0 – 119.6) | 119.1 (118.2 – 119.6) | 119.1 (118.2 – 119.6) |
