# Supplementary material for: Linking Light-Dependent Life History Traits with Population Dynamics for Prochlorococcus and Cyanophage
Source: mSystems. 2020 Mar 31;5(2):e00586-19. doi: 10.1128/mSystems.00586-19 (PMC7112961; doi:10.1128/mSystems.00586-19)
Supplement: TEXT S1 [file msystems.00586-19-s0001.pdf]

We tested different hypotheses and built candidate models based on the initial model that can explain the lysis slowdown. Here we listed the candidate models that reproduced a lysis slowdown and represent their dynamics in the SI Fig 5.

Candidate model 1: different eclipse and lysis periods

We used the initial model (Eq. 5) and subdivided latent period into two periods: the eclipse period λ_e and the lysis period λ_l such that $\lambda = \lambda_e + \lambda_l$. The dynamics of the candidate model 1 are represented with cyan solid lines in the SI Fig 5.

Candidate model 2: lysis decrease with viral concentration

The candidate model 2 is similar to the initial model (Eq. 5) but the latent period is now a function of the viral concentration as follows:

$$\lambda = \lambda_0 MOI \frac{V}{N}. \tag{1}$$

where λ_0 is the basal latent period and MOI is the MOI rate of the treatment ($MOI = 0.1$). The dynamics of the candidate model 2 are represented with green solid lines in the SI Fig 5.

Candidate model 3: host cells mutation lead to resistance to infection

The candidate model 3 is based on the initial model (Eq. 5) but allows the host cell S to mutate at a rate r and become resistant to infection. The resistant host cells R grow at a lower rate than S, given a presumed fitness cost to resistance:

$$\frac{dR}{dt} = \epsilon\mu R(1 - \frac{N}{K}) + rS - \omega R, \tag{2}$$

where ϵ is the cost of resistance ($\epsilon = 0.5$), N is the total host population such that $N = S + E + I + R$ and r is the mutation rate from the class S to R. We used the mutation rate measured by [68] for *Prochlorococcus* strain MED4 and consider that cell divide ones per day ($r = 6.08\ 10^{-6}$ per division). The dynamics of the candidate model 3 are represented with pink solid lines in the SI Fig 5.

Candidate model 4: unsuccessful adsorption

The candidate model 4 is based on the initial model (Eq. 5) but we assume that adsorption does not necessarily lead to infection. Instead, viruses V can adsorb at a rate ϕ to susceptible cells S which leads to a new infection in an ϵ fraction of cases. The dynamics of candidate model 4 are represented with red solid lines in the SI Fig 5.
